# Emerging Role for the PERK/eIF2α/ATF4 in Human Cutaneous Leishmaniasis

**DOI:** 10.1038/s41598-017-17252-x

**Published:** 2017-12-06

**Authors:** Karina Luiza Dias-Teixeira, Teresa C. Calegari-Silva, Jorge M. Medina, Áislan C. Vivarini, Átila Cavalcanti, Nataly Teteo, Alynne Karen M. Santana, Fernando Real, Ciro M. Gomes, Renata Meirelles Santos Pereira, Nicolas Fasel, João S. Silva, Bertal H. Aktas, Ulisses G. Lopes

**Affiliations:** 1 Laboratory of Molecular Parasitology, Institute of Biophysics - Federal University of Rio de Janeiro, Rio de Janeiro, RJ 21949-902 Brazil; 2 Hematology Laboratory for Translation, Department of Medicine, Brigham and Women’s Hospital and Harvard Medical School, Boston, MA 02115 United States; 3 Institute of Medical Biochemistry, Federal University of Rio de Janeiro, Rio de Janeiro, RJ 21949-902 Brazil; 4 Department of Biochemistry and Immunology - University of São Paulo, Ribeirão Preto, SP 14049-900 Brazil; 5 Department of Microbiology, Immunology and Parasitology, Federal University of São Paulo, São Paulo, SP 04023-062 Brazil; 6 Faculty of Medicine, University of Brasília, Brasília, DF 70910-900 Brazil; 7 Instituto de Microbiologia Paulo de Goes, Federal University of Rio de Janeiro, 21942-902 Rio de Janeiro, Brazil; 8 Center for Immunity and Infection Lausanne, Department of Biochemistry, Faculty of Biology and Medicine, University of Lausanne, CH-1066 Epalinges, Switzerland

## Abstract

*Leishmania* parasites utilize adaptive evasion mechanisms in infected macrophages to overcome host defenses and proliferate. We report here that the PERK/eIF2α/ATF4 signaling branch of the integrated endoplasmic reticulum stress response (IERSR) is activated by *Leishmania* and this pathway is important for *Leishmania amazonensis* infection. Knocking down PERK or ATF4 expression or inhibiting PERK kinase activity diminished *L*. *amazonensis* infection. Knocking down ATF4 decreased NRF2 expression and its nuclear translocation, reduced HO-1 expression and increased nitric oxide production. Meanwhile, the increased expression of ATF4 and HO-1 mRNAs were observed in lesions derived from patients infected with the prevalent related species *L*.*(V*.*) braziliensis*. Our data demonstrates that *Leishmania* parasites activate the PERK/eIF2α/ATF-4 pathway in cultured macrophages and infected human tissue and that this pathway is important for parasite survival and progression of the infection.

## Introduction

Human leishmaniasis is endemic in nearly 100 countries and affects ~1.5 million people in its cutaneous or visceral forms. Leishmaniasis is also an important infection associated with HIV^1^. *Leishmania amazonensis* is endemic in north and central Brazil and is an important etiological agent of human cutaneous leishmaniasis^2^. How these parasites avoid the host immune system, particularly how they subvert innate immune responses to establish a successful infection is the subject of intense scientific scrutiny^3^. A body of evidence indicates that *Leishmania* parasites activate and subvert the classical host antiviral defenses to promote survival. For example, *L*. *amazonensis* parasites induce IFN1-β and IL-10 expression via PKR activation in a Toll-like receptor 2 (TLR2) dependent manner, a prototypical component of cellular innate immunity^4,5^. The integrated endoplasmic reticulum stress response (IERSR) may have functioned as a primitive immune defense for unicellular organisms^6^. In mammalian cells, the IERSR has evolved as a three-pronged response involving activating transcription factor 6 (ATF6), inositol-requiring enzyme 1 (IRE1) and PKR- like endoplasmic reticulum kinase (PERK)^6^. ATF6 is a proteolytically activated transcription factor that plays critical roles in expanding the folding capacity of the ER and ER-associated retrograde transport and degradation (ERAD) of misfolded proteins. IRE1, through its downstream effector, the X-box binding protein 1 (XBP-1), induces a cluster of genes to expand the folding capacity of the ER and promote ERAD^6–9^. The third arm of the IERSR is the PERK-dependent phosphorylation of eukaryotic initiation factor 2α (eIF2α), which inhibits translation initiation to reduce the demand on the folding capacity of the ER while paradoxically upregulating the translation of a small subset of mRNAs, including that of ATF4 mRNA. ATF4 induces transcription of a group of genes, including its own, that play critical roles in ERAD, autophagy and apoptosis^10^. PERK also phosphorylates and activates the nuclear factor (erythroid-derived 2)-like 2 (NRF2), which can coordinate with ATF4 to induce expression of anti-oxidative genes^11–13^.

The PERK/eIF2α/ATF4 axis of the IERSR plays a critical role in cell survival under various stress conditions, including genotoxic, nutritional, hypoxic and oxidative stress^10^. Infection by intracellular pathogens induces host cell stress responses, as exemplified by activation of eIF2α phosphorylation by viral infection^14^. By hijacking this important pathway, intracellular parasites may facilitate the survival of host cells for their own maximal proliferation and protect themselves from the harmful consequences of cellular stress, including the shutdown of metabolic pathways and accumulation of reactive oxygen species (ROS). Shutdown of host protein synthesis frees up ribosomes to produce large quantities of viral proteins^14^. We and others have previously shown that XBP-1 is activated by intracellular protozoan parasites and that this protein plays a significant role in infection^15,16^. Given the coordinated activation of XBP-1 and ATF4 under cellular stress, we hypothesized that *L*. *amazonensis* and *Leishmania (V*.*) braziliensis* species could also activate the PERK/eIF2α/ATF4 signaling axis and that this pathway would play a critical role in intracellular survival and in cutaneous leishmanisis (CL) pathogenesis^17^. We report here in that PERK/eIF2α/ATF4 branch of the IERSRS is activated *in vitro* as well as in infected tissues of CL human patients, and that this pathway is important for successful establishment of *Leishmania* infection. We further report that PERK/eIF2α/ATF4 signaling promotes parasite survival and proliferation.

## Methods

### Culture of mammalian cells

The human embryonic kidney cell line HEK-293FT (Thermo Scientific-Life Technologies, Waltham, MA, USA) was cultured in DMEM (Thermo Scientific-Gibco, Grand Island, NY, USA) supplemented with 10% heat-inactivated fetal bovine serum (GIBCO) with 100 U/mL penicillin and 100 µ/mL streptomycin at 37 °C in 5% CO_2_. The HEK293FT cells were maintained with 500 μg/mL of geneticin (SIGMA ALDRICH, St. Louis, MO, USA). The murine macrophage cell line RAW264.7 (ATCC:TIB-71, American Type Culture Collection, Manassas, VA, USA) was cultured in DMEM (GIBCO) supplemented with 10% heat-inactivated fetal bovine serum (GIBCO) with 100 U/mL penicillin and 100 µ/mL streptomycin at 37 °C in 5% CO_2_. The human monocytic leukemia cell line THP-1 (ATCC:TIB202TM) was cultured in RPMI (GIBCO) supplemented with 20% heat-inactivated fetal bovine serum (GIBCO) with 100 U/mL penicillin and 100 mg/mL streptomycin at 37 °C in 5% CO_2_.

Murine primary macrophages were thioglycollate-elicited and removed from wild-type (WT) or TLR4-knockout (KO) C57BL/6 mice by peritoneal washing. Primary macrophages were dispensed into 6-well plates at a 2 × 10^6^ concentration for 1 hour to allow cells to adhere. Adherent cells were cultured for an additional 24 hours in DMEM.

(GIBCO) supplemented with 10% heat-inactivated fetal bovine serum (GIBCO) with 100 U/mL of penicillin and 100 µ/mL streptomycin at 37 °C in 5% CO_2_.

### Culture of *Leishmania* and infection

*Leishmania amazonensis* (WHOM/75/Josefa) promastigotes were cultured in Schneider insect medium (SIGMA-ALDRICH) supplemented with 10% heat-inactivated fetal bovine serum, 100 U/mL penicillin and 100 mg/mL streptomycin at 26 °C. Parasites were transferred to fresh medium when they reached the density of 10^7^ parasites/mL.

RAW 264.7, THP-1 or primary macrophages were infected with stationary promastigotes (4–5 days) at a ratio of 10 parasites per macrophage. The infection index was estimated by multiplying the percentage of infected macrophages by the average of parasite number per macrophage on Giemsa-stained slides (Accustain^®^ modified Giemsa, SIGMA-ALDRICH). The number of infected macrophages and the average number of parasites per macrophage was determined in 300 cells in each experiment.

### Cell treatment

As a positive control for ER stress induction, we treated the cells with vehicle (DMSO) (SIGMA-ALDRICH) or 1 μM Thapsigargin (TG) (SIGMA-ALDRICH) for 1 or 8 hours. To determine the role of PERK in *L*. *amazonensis* infection, we treated the cells with vehicle (DMSO) or PERK inhibitor (GSK2606414 - MERCK) for 1 hour before infection. To determine the role of oxidative stress in our model, we treated the cells with 20 mM of N-acetyl-cysteine (NAC) (SIGMA- ALDRICH) for 30 minutes, infected the culture with *L*. *amazonensis* for 24 hours, and treated the infected culture again with 20 mM of NAC for additional 48 hours. For the luciferase assay, we used 10 μM sulforaphane (SIGMA) for 24 hours as a positive control for oxidative stress induction. As a positive control to NRF2 activation, we used 100ng/mL LPS (SIGMA-ALDRICH).

### Immunoblotting analysis

RAW 264.7 cells or primary murine macrophages were dispensed in 6-well polystyrene plates at a concentration of 10^6^ cells/mL (RAW 264.7) or 2 × 10^6^ cells/mL (primary murine macrophages) one day before infection. Protein extracts were obtained from cells infected with *L. amazonensis* for 1, 4 and 8 hours. For whole cell lysates, cells were washed 3 times with PBS buffer and subsequently lysed in 0,1 mL of lysis buffer (50 mM Tris-HCl (pH 7.5), 250 mM NaCl, 5 mM EDTA, 10 mM EGTA, 50 mM NaF, 20 mM β-glycerophosphate, 0.1% Triton X-100 and 1 μg/mL bovine serum albumin) supplemented with a cocktail of protease and phosphatase inhibitors (SIGMA-ALDRICH). Nuclear extracts were obtained as described^18^. Whole cell lysates (50 μg) and nuclear extracts (20 μg) were subjected to SDS-PAGE and transferred to PVDF membranes (Bio-Rad, Hercules, CA, USA). Membranes were incubated for 12 hours with commercial antibodies against phospho-PERK (Cell Signaling, Danvers, MA), HO-1 (Stressgen Biotechnologies, Victoria, BC, Canada), phospho-eIF2α (Epitomics), iNOS (Cell Signaling), NRF2 (Cell Signaling), α-tubulin (Cell Signaling), β-actin (SIGMA-ALDRICH) lamin A/C and HSP70 (Santa Cruz Biotechnology, Dallas, TX, USA). Membranes were then incubated with horseradish peroxidase-conjugated antibody (1:4000) for 1 hour at room temperature and washed 3 times with tris-buffered saline with 0.01% of tween (TBS-T). Proteins were detected with the ECL chemiluminescent detection system (GE Health Care, Pittsburgh, PA, USA).

### RT-qPCR

Total RNA was extracted from *in vitro* infected macrophages, peritoneal macrophages or skin biopsies of patients infected with *L*. *braziliensis* and from skin samples of healthy patients and reverse-transcribed into first-strand cDNA using the ImProm-II^TM^ Reverse Transcription System (PROMEGA, Madison WI, USA). The following primer pairs were used to determine the mRNA levels for ATF4, XBP-1, HO-1 and GAPDH: forward 5′-TCTCATTCAGGCTTCTCACGGCAT-3′ and reverse 5′-AAGCTCATTTCGGTCATGTTGCGG-3′ primers were used to amplify ATF4 mRNA; forward 5′-AGCACTCAGACTACGTGCACCTCT-3′ and reverse 5′-GAAGAGTCAATACCGCCAGAATCC-3′ primers were used to amplify uXBP-1 mRNA; forward 5′-TGCTGAGTCCGCAGCAGGT-3′ and reverse 5′-CCAGAATGCCCAACAGGATATCAG-3′ primers were used to amplify sXBP-1 mRNA; forward 5′-GCAGAGAATGCTGAGTTCATG-3′ and reverse 5′-CCTCCTCCAGGGCCACATAGATGTG-3′ primers were used to amplify HO-1 mRNA; forward 5′-CTCCCAATTCAGCCGGCCC-3′ and reverse 5′-CCAGGGCAAGCGACTCATGG-3′ primers were used to amplify NRF2 mRNA; forward 5′-TGCACCACCAACTGCTTAGC-3′ and reverse 5′-GGCATGGCATGTGGTCATGAG3′ primers were used to amplify GAPDH mRNA. GAPDH was used as an endogenous control to normalize expression levels of all other transcripts. Melting curves were analyzed for the presence of a single melting temperature to determine amplicon specificity. The RT-qPCR assays were performed on an Applied Biosystems 7500 using Power SYBR Green Master Mix (Thermo Scientific-Applied Biosystems, Foster City, CA, USA). All expression ratios were processed *via* the delta-delta Ct method.

### Immunofluorescence

RAW 264.7 cells (2 × 10^5^) were plated in a 24 well-plate and infected for 8 hours with *L*. *amazonensis*. After infection, cells were fixed for 10 minutes with 4% paraformaldehyde and processed for immunofluorescence as follows: cells were permeabilized with 50 μg/mL digitonin in PBS for 5 minutes at room temperature, blocked with 1% bovine serum albumin (BSA, SIGMA-ALDRICH) in PBS for 1 hour at room temperature and then incubated overnight with anti-rabbit ATF4 polyclonal antibody (Cell Signaling) followed by incubation with an AlexaFluor 568-conjugated anti-rabbit secondary antibody (Thermo Scientific). DAPI was used for staining host and parasite DNA. Images were acquired with an LSM 780 multiphoton microscope and processed using ICY and ImageJ software.

### Luciferase assay

To analyze the ATF4-dependent transcriptional activity, shSCR or shATF4 transduced cells were plated at concentrations of 10^5^ cells/mL in a 48 well-plate and transfected with Lipofectamine 2000 (Thermo Scientific). For transfections, 1 μg of pGL23xARE-LUC, 1 μg of pGL2NRF2-LUC and 40 ng pRL-CMV (PROMEGA) were used.

Transfected cells were incubated for 24 hours and then infected with *L*. *amazonensis* (10:1) for an additional 24 hours. After infection, cells were washed with PBS, lysed according to the Dual Luciferase System protocol (PROMEGA) and analyzed using a Glomax luminometer (PROMEGA). Sulforaphane treatment was used as a positive control of Nrf2- Luciferase assyas.

### Lentiviral transduction

PERK and ATF4 were knocked down in RAW264.7 cells by transducing cells with lentiviral PERK shRNA or an ATF4 shRNA expression vector as described^19^. The mouse shPERK and shATF4 plasmids vectors were obtained from the Dana Farber Institute/Broad Institute shRNA Consortium (Boston, MA, USA). The pLKO plasmid, the pMD2G envelope plasmid and the PSPAX2 packaging plasmid were obtained from Addgene (Cambridge MA, USA). Cells were selected three days after transduction procedure with 4 μg/mL of puromycin for 7 days.

### Determination of Nitric Oxide production

To determine the nitric oxide concentration, we proceeded with the Griess reaction to analyze the nitrite content in the cell supernatant as an indicator of NO production by scrambled shRNA or shATF4 cells infected by *L*. *amazonensis* (10:1). 50 μL of culture supernatant was mixed with 50 μL of a solution containing N-[naphthyl] ethylenediamine dihydrochloride (NEED; 1 mg/mL), sulfanilamide (10 mg/mL) and 5% phosphoric acid. The absorbance was measured by spectrophotometry at 540 nm.

### Immunohistochemistry (IHC)

Biopsy specimens from lesion borders of patients with cutaneous leishmaniasis and from healthy donors were frozen in optimal-cutting-temperature compound (Sakura Finetek, Torrance, CA), sectioned (5 µm) in Cryostate HM 525 (Microm-Zeiss, Germany) and fixed in ice-cold acetone before incubation with anti-human ATF4 (1:100) antibody (Abcam, Cambridge, MA). The slides were counterstained with Harris’s Hematoxylin (Millipore, USA), dried, and mounted with Permout (Millipore, USA). Analyses were performed by two independent observers, to avoid intraobserver bias. Photomicrographs (×40) were obtained by light microscope with the AxioVision Imaging System A1 (Zeiss, Germany).

### Statistical analysis

Whenever two groups of data were considered, data were analyzed by Student’s t test for independent samples. Comparisons involving more than two groups were performed by one-way-ANOVA followed by Bonferroni post hoc test. Data are expressed as bars of mean ± standard deviation (SD) from pools of at least 3 experiments. Experiments with patient derived samples were analyzed with Mann-Whitney non-parametric test and their data are represented as median with amplitude bars. All analysis were conducted using Prism 6 Software (Graph Pad).

### Approval Statement

The methods carried out in this work are in accordance with the guidelines approved by the Ethical Committee of Biological Research Experimentation, Federal University of Rio de Janeiro, Brazil. Experimental protocols using cells derived from mice were approved by the Federal University of Rio de Janeiro Committee for Animal Use (permit numbers: IMPPG 024 and IBCCF 171). For experimental protocols using material from human biopsies, patients were selected from a cohort study following the Ethics Committee approval from the Faculty of Medicine - University of Brasilia (permit number 35611714.7.1001.5558).

### Accordance Statement

The experiments were carried out in accordance with the National and Academic guidelines and regulation from each Institution enrolled in the present study.

### Informed Consent Statement

An informed consent was obtained from all participants and/or their legal guardian/s regarding the utilization of human tissues samples for IH studies.

## Results

### The PERK/eIF2α IERSR signaling is induced by *L*. *amazonensis* and favors parasite infection

**Figure 1:**
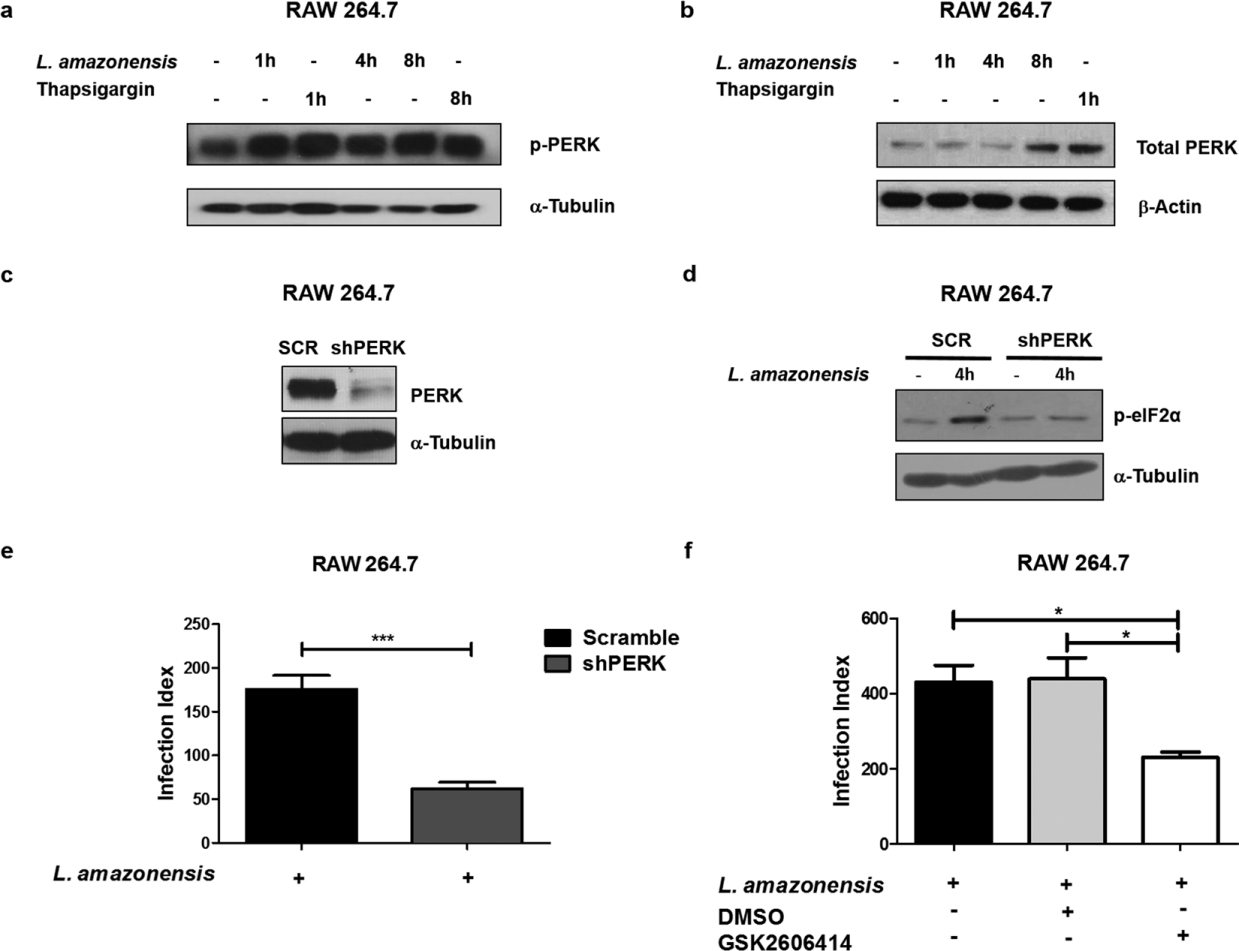
*L*. *amazonensis* induces PERK/eIF2α activation and depletion of PERK reduces infection. (**a**) RAW 264.7 cells were infected with *L*. *amazonensis* for 1, 4 and 8 hours and the total protein extract was analyzed by western blot with anti phospho-PERK and α-tubulin antibodies. RAW 264.7 were treated with 1 µM of thapsigargin for 1 and 8 hours as a positive control. (**b**) RAW 264.7 cells were infected with *L. amazonensis* for 1, 4 and 8 hours and the total protein extract was analyzed by western blot with anti total- PERK and b-actin antibodies. RAW 264.7 were treated with 1 µM of thapsigargin for 1 hour as a positive control. (**c**) Extracts prepared from RAW 264.7 cells transduced with shSCR or shPERK expression vectors were analyzed by western blot with anti-total-PERK and α-tubulin antibodies. (**d**) RAW 264.7 transduced with shPERK or shSCR expression vectors were infected with *L*. *amazonensis* for 4 hours and cell lysates were analyzed by western blot with anti-phospho-eIF2α and α-tubulin antibodies. (**e**) shSCR and shPERK transduced RAW 264.7 cells were infected with *L. amazonensis* for 48 hours, fixed and stained with Giemsa. Infection Index was measured as percent of infected cells × number of the amastigotes/cell. ***Indicates p < 0.001. Bars represent mean ± SD from 3 independent experiments. (**f**) RAW 264.7 cells were treated with PERK inhibitor (GSK2606414) or vehicle (DMSO) and infected with *L*. *amazonensis* for 48 hours. After 48 hours, cells were fixed and stained with Giemsa. Infection Index was measured as in (**e**). *Indicates p < 0.05. Bars represent mean ± SD from 3 independent experiments. Representative western blots are shown from 3 independent experiments (**a**,**c**,**d**) and from 3 independent experiments (**b**).

To test the hypothesis that *Leishmania* parasites are able to activate the PERK/eIF2α/ATF4 branch of the IERSR, we infected RAW264.7 mouse macrophage cell line with *L*. *amazonensis* parasites and analyzed total cell extracts for phosphorylated PERK by western blot (Fig. 1a). We observed that *L*. *amazonensis* infection induces PERK phosphorylation, which is indicative of activation of this kinase and increased the levels of total PERK after 8 hours of infection (Fig. 1b) similarly to the cells treated with thapsigargin for 1 hour. To determine the importance of PERK/eIF2α/ATF4 for *L*. *amazonensis* infection, we transduced mouse macrophages with lentiviral short hairpin (sh) RNA expression vectors targeting PERK (shPERK) or scrambled shRNA (shSCR) and selected stably transduced cells. Figure 1c shows that shPERK transduced macrophages express dramatically less PERK compared to shSCR transduced macrophages. eIF2α is the best-known substrate of PERK and its phosphorylation attenuates mRNA translation^10^. We therefore infected shPERK and shSCR transduced macrophages with *L*. *amazonensis* for 4 hours and analyzed the levels of phosphorylated eIF2α (p-eIF2α) by western blot (Fig. 1d). We observed a significant reduction of p-eIF2α in *L*. *amazonensis* infected shPERK-transduced cell lysates compared to shSCR transduced lysates, indicating that *L*. *amazonensis* induces eIF2α phosphorylation in a PERK dependent manner. To determine if PERK is essential for *L*. *amazonensis* infection, we compared the infection index in shSCR or shPERK transduced macrophages. As shown in Fig. 1e, knocking down PERK expression significantly reduces the infection index in about 65% compared to scramble transfected cells. We observed that shScramble cells and shPERK cells infected with *L*. *amazonensis* for 4 hours display similar parasite load, which indicates that the role of PERK in *L. amazonensis* infection (Supplementary Figure 1a) does not rely on reduced parasite uptake. To confirm the finding that PERK is essential for *L*. *amazonensis* infection, we treated parental RAW264.7 cells with vehicle (DMSO) or with a well characterized PERK inhibitor (GSK2606414). Then, we infected the cells with *L*. *amazonensis* for 48 hours and measured the infection index. The PERK inhibitor significantly reduces *L*. *amazonensis* infection when compared to DMSO (Fig. 1f) supporting our findings that knocking down PERK expression reduces *L*. *amazonensis* infection. Taken together, these data demonstrate that *L*. *amazonensis* induces the PERK/eIF2α signaling axis, and that this pathway plays a critical role in the infection.

### *L*. *amazonensis* infection induces ATF4, a downstream effector of the PERK/eIF2α branch of the IERSR

Activation of eIF2α by PERK leads to the inhibition of the translation initiation. Nevertheless, eIF2α phosphorylation upregulates translation of a small subset of mRNAs^20^. Specifically, mRNAs with multiple tandem upstream open reading frames (uORF) in their 5′ untranslated region (5′UTR), such as the mRNA coding for ATF4, are translated more efficiently when eIF2α is phosphorylated^21,22^. Therefore, expression of ATF4 is increased in response to eIF2α phosphorylation, which in turn activates transcription of a number of genes involved in ER homeostasis, autophagy and oxidative stress response^10–12^.

**Figure 2:**
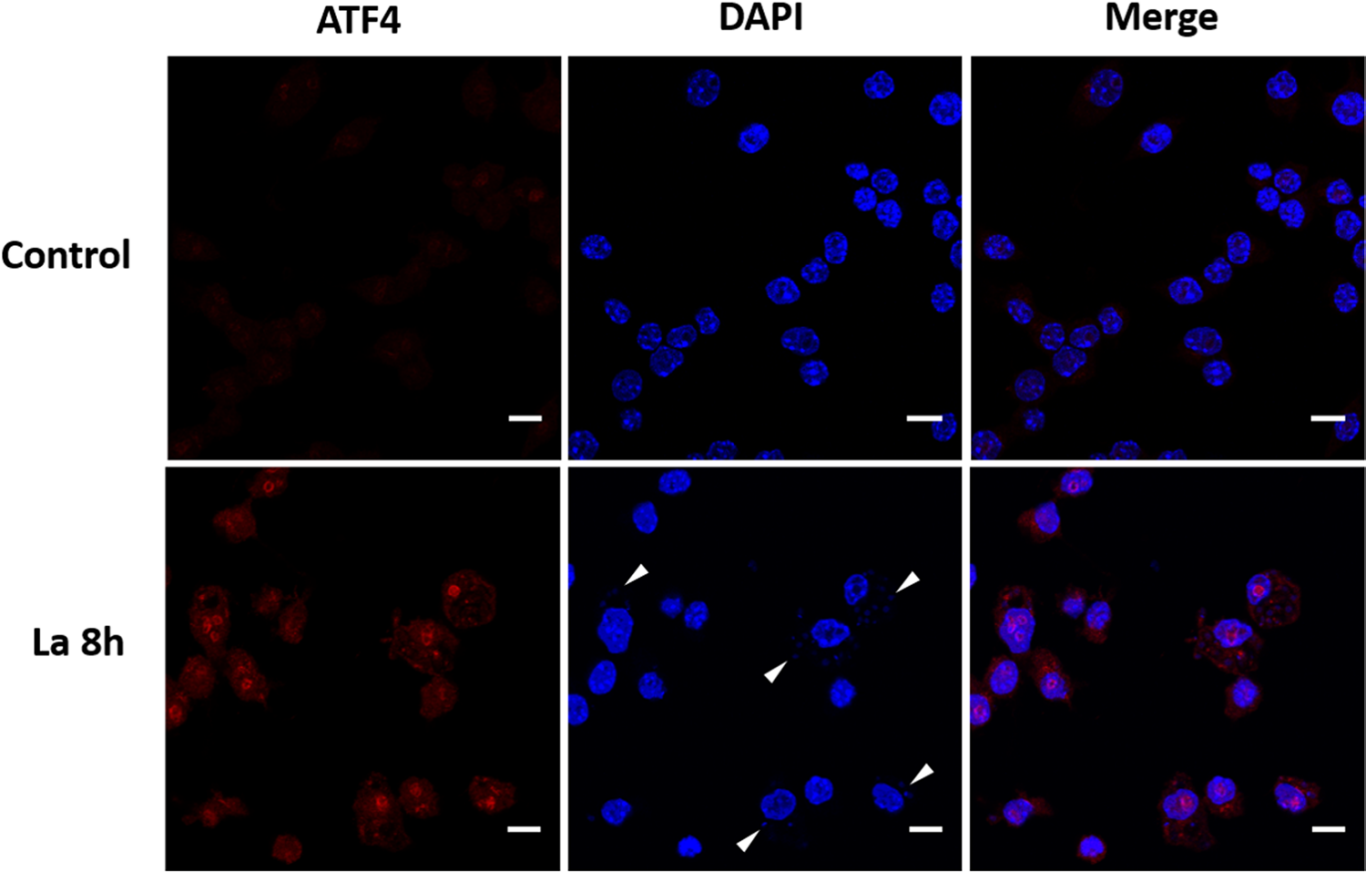
ATF4 is expressed and translocated to the cell nucleus during *L*. *amazonensis* infection. RAW 264.7 cells were plated and infected with *L*. *amazonensis* for 8 hours. Cells were fixed, incubated with anti-ATF4 antibody and Alexa 568 labeled secondary antibody. DAPI was used for host and parasite DNA staining. White arrowheads point to stained nucleus/kinetoplast of amastigotes. Images were acquired with multiphoton microscopy. Scale bar: 10 μm.

**Figure 3:**
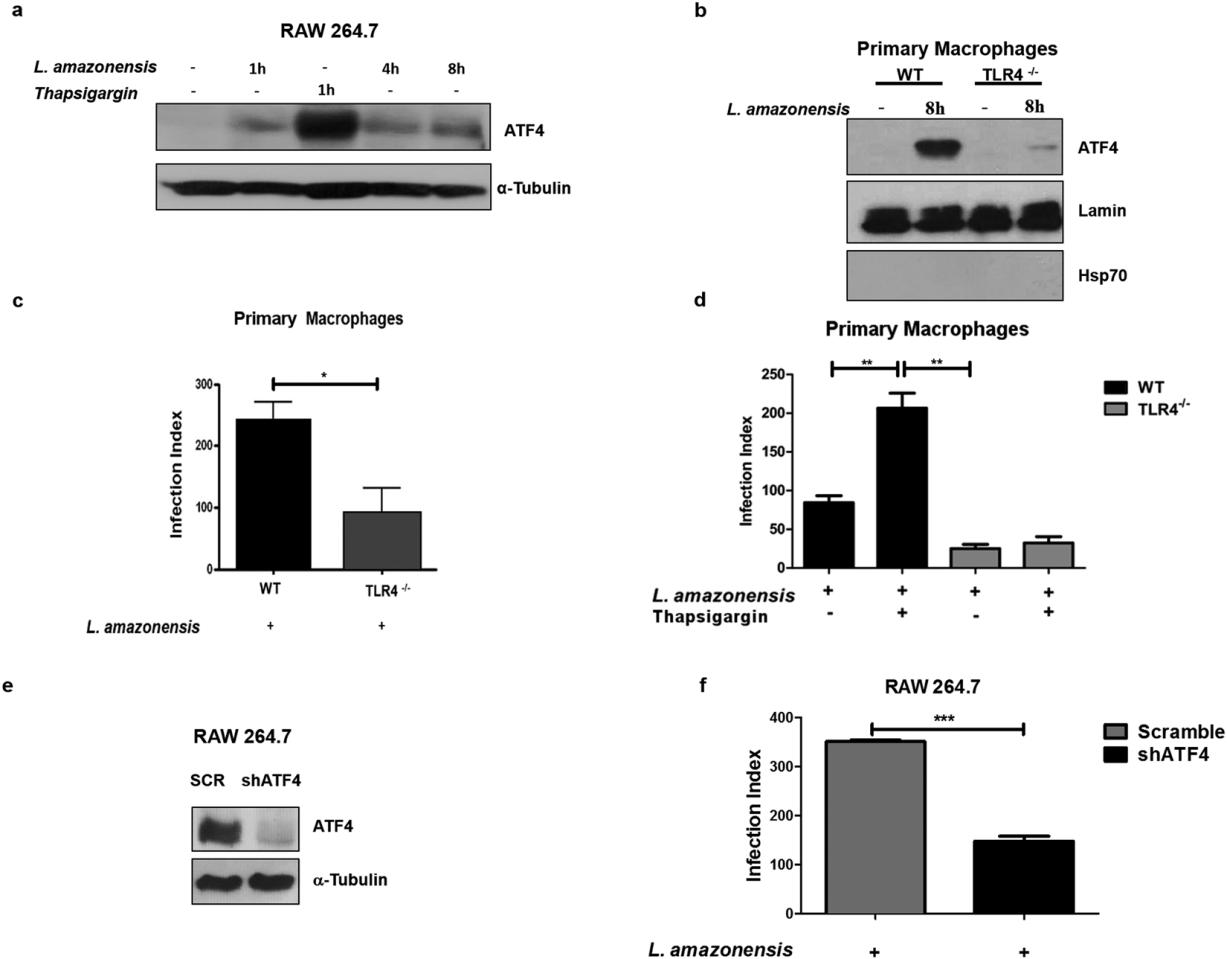
*L*. *amazonensis* induces ATF4 activation and its depletion reduces *L. amazonensis* infection. (**a**) RAW 264.7 cells were infected with *L. amazonensis* for 1, 4 and 8 hours respectively treated with 1 µM of thapsigargin for 1 hour as a positive control. The total protein extract was analyzed by western blot with anti-ATF4 and α-tubulin antibodies. (**b**) Primary macrophages from wild-type or TLR4 knockout mice were infected with *L. amazonensis* for 8 hours and the nuclear extracts were analyzed by western blot with anti-ATF4, lamin a/c and hsp70 antibodies. (**c** and **d**) Primary macrophages from wild-type or TLR4 knockout mice were infected with *L. amazonensis* for 48 hours and treated or not with thapsigargin (1 mM), fixed and stained with Giemsa. Infection Index was measured as in Fig. 1d. (**e**) RAW 264.7 transduced with shSCR or shATF4 expression vectors were treated for 1 hour with thapsigargin and the total extract was obtained and analyzed by western blot with anti-ATF4 and α-tubulin antibodies. (**f**) shSCR and shATF4 cells were infected with *L. amazonensis* for 48 hours, fixed and stained with Giemsa. Infection Index was measured as in Fig. 1d. *Indicates p < 0.05. **Indicates p < 0.01. ***Indicates p < 0.001. Bars represent mean ± SD from 3 independent experiments. Representative western blots are shown from 3 independent experiments (**a**–**c**).

To understand whether the activation of PERK/eIF2α by *L*. *amazonensis* parasites induces ATF4 expression, we obtained whole cell lysates from RAW264.7 cells infected with *L*. *amazonensis* for 1, 4 and 8 hours and determined ATF4 expression by western blot analysis. Consistent with activation of PERK and eIF2α phosphorylation, *L*. *amazonensis* increased ATF4 expression and cause ATF4 nuclear translocation when compared with uninfected cells (Figs 2 and 3a).

Others have reported that ATF4 can be recruited by TLR4 to regulate cytokine production^23^ while it has been described that *Leishmania* belonging to the Mexicana complex express TLR4 ligands^24,25^. To determine if TLR4 was required for ATF4 induction in infected macrophages, we prepared nuclear extracts from wild-type and TLR4 knockout murine cells infected with *L*. *amazonensis* for 8 hours. As shown in Fig. 3b, infection of TLR4-KO macrophages led to a remarkable reduction of ATF4 nuclear translocation when compared with infected wild-type cells. Infection Index assays showed that TLR4 also plays a role in *L*. *amazonensis* infection (Fig. 3c and d).

To determine the role of ATF4 in *L*. *amazonensis* infection, we generated ATF4 knockdown macrophages by transducing RAW-264.7 cells with lentiviral vector targeting ATF4. Efficiency of the ATF4 knockdown in transduced cells was more than 80%, as shown in Fig. 3e. Using shSCR cells as control, we assayed parasite burden in shATF4 transduced macrophages. Analysis of infection index demonstrated that ATF4 knockdown cells significantly reduces *L*. *amazonensis* burden in about 58% compared to scramble transfected cells (Fig. 3f). We observed that shScramble cells and shATF4 cells infected with *L*. *amazonensis* for 4 hours display similar parasite load, which indicates ATF4 knockdown does not reduce the parasite uptake (Supplementary Figure 1b). Taken together, these results delineate a context in which *L*. *amazonensis* induces the PERK/eIF2α/ATF4 signaling pathway and demonstrate that this mechanism plays a critical role in *L*. *amazonensis* infection.

### PERK/eIF2α/ATF4 signaling protects *L*. *amazonensis-*infected macrophages from oxidative stress

**Figure 4:**
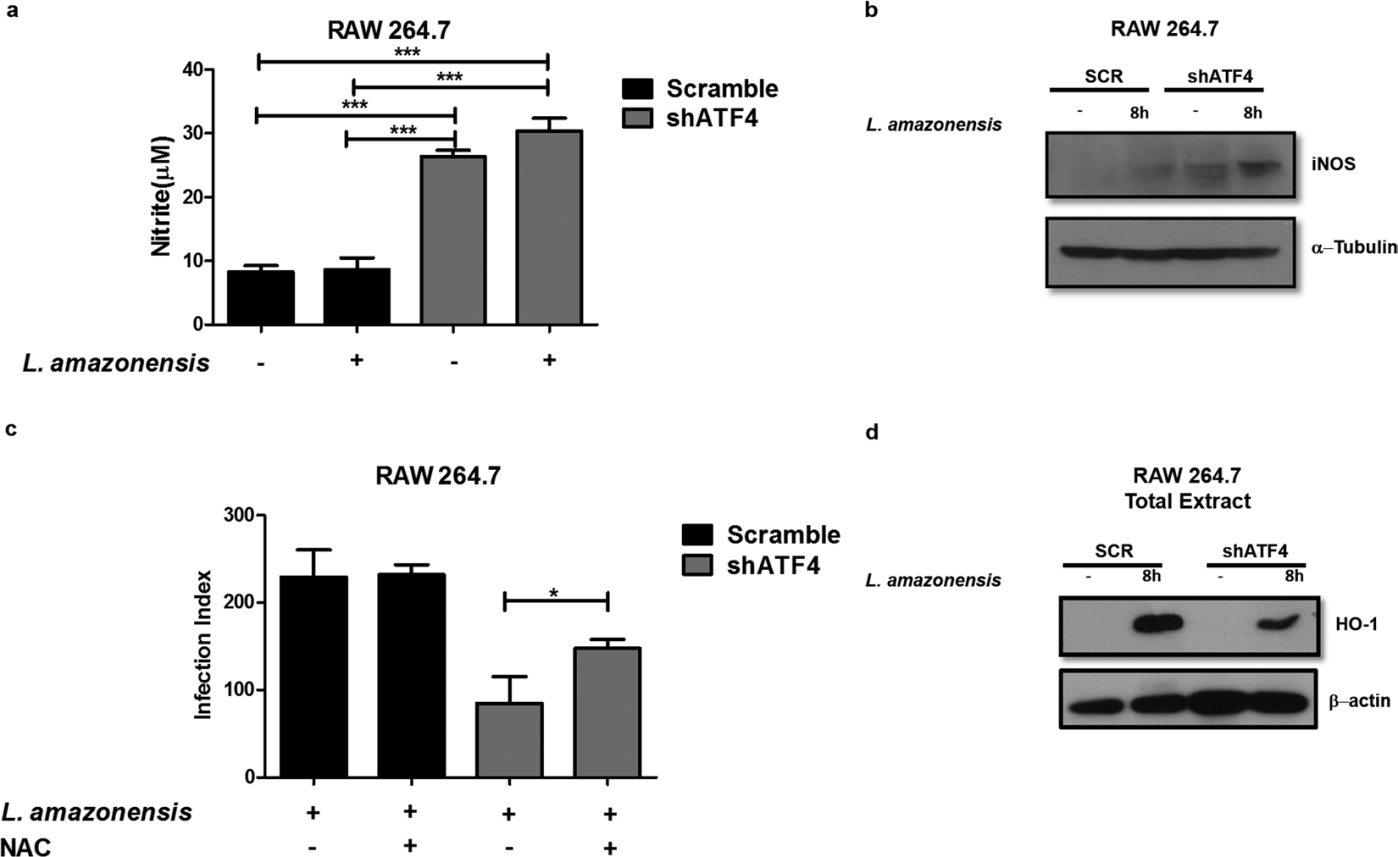
ATF4 protects *L*. *amazonensis* from host oxidative stress. (**a**) shATF4 or shSCR transduced cells were infected with *L*. *amazonensis* for 48 hours and nitrite concentration was measured by Griess reaction. ***Indicates p < 0.001. Bars represent mean ± SD from 3 independent experiments. (**b**) shATF4 or shSCR transduced cells were infected with *L*. *amazonensis* for 24 hours and the total extract was obtained and analyzed by western blot with anti-iNOS and α−tubulin antibodies. (**c**) shATF4 or shSCR transduced cells were infected with *L*. *amazonensis* for 24 hours and treated with NAC for 24 hours. Cells were fixed, stained with Giemsa and infection indexes were measured as in Fig. 1d. ***Indicates p < 0.001. Bars represent mean ± SD from 3 independent experiments. (**d**) shATF4 or shSCR transduced cells were infected with *L. amazonensis* for 8 hours and total cell lysates were probed with anti-HO-1 and anti-β- actin specific antibodies. Representative western blot are shown from 3 independent experiments (**b**,**d**).

At the molecular level, ATF4 plays a critical role in protection from oxidative stress. ATF4 can form dimers with the redox-sensitive transcription factor NRF2 and control response to the oxidative stress following ER stress through the expression of HO-1^11–13,26^. To determine whether ATF4 promotes *L*. *amazonensis* infection by inducing oxidative stress defense, we evaluated the role of ATF4 in controlling the oxidative stress response during infection. We infected shSCR or shATF4 transduced macrophages with *L*. *amazonensis* for 24 hours and evaluated nitrite production as an indirect measure of nitric oxide (NO) levels. We observed that infected shATF4- macrophages have increased NO production (Fig. 4a). Western blot analysis of whole cell lysates obtained from infected shATF4 or shSCR-transduced macrophages showed that knocking down ATF4 expression increases iNOS expression (Fig. 4b). To determine the functional relevance of these observations, we treated *L*. *amazonensis* infected shATF4 or shSCR transduced macrophages with N-acetyl-cysteine (NAC, a well-characterized anti-oxidant agent) or vehicle and determined infection indexes. Our data demonstrate that NAC effectively counteracted the reduction of the infection index in shATF4 macrophages (Fig. 4c). These data demonstrate that PERK/eIF2α/ATF4 signaling axis is an important *Leishmania* pro-survival element due to its regulator role in the anti-oxidative cell response.

### ATF4 is critical for NRF2 and HO-1 expression in *L*. *amazonensis* infected macrophages

ATF4 regulates gene expression of anti-oxidant gene products, such as heme oxygenase (HO-1)^11,26^. We show that shATF4-transduced macrophages infected with *L*. *amazonensis* express much lower levels of heme oxygenase enzyme (Fig. 4d). ATF4 promotes protection from oxidative stress by forming a transcriptionally active complex with NRF2, an important transcription factor involved in the regulation of the oxidative stress response^26^. Although there are no canonical binding sites for ATF4 in the heme oxygenase promoter, HO-1 gene is a known target of NRF2. Moreover, there are ARE elements in the HO-1 promoter, and ATF4 and NRF2 may form heterodimer/multimers to induce the expression of HO-1 by binding one or more of these elements^26^.

**Figure 5:**
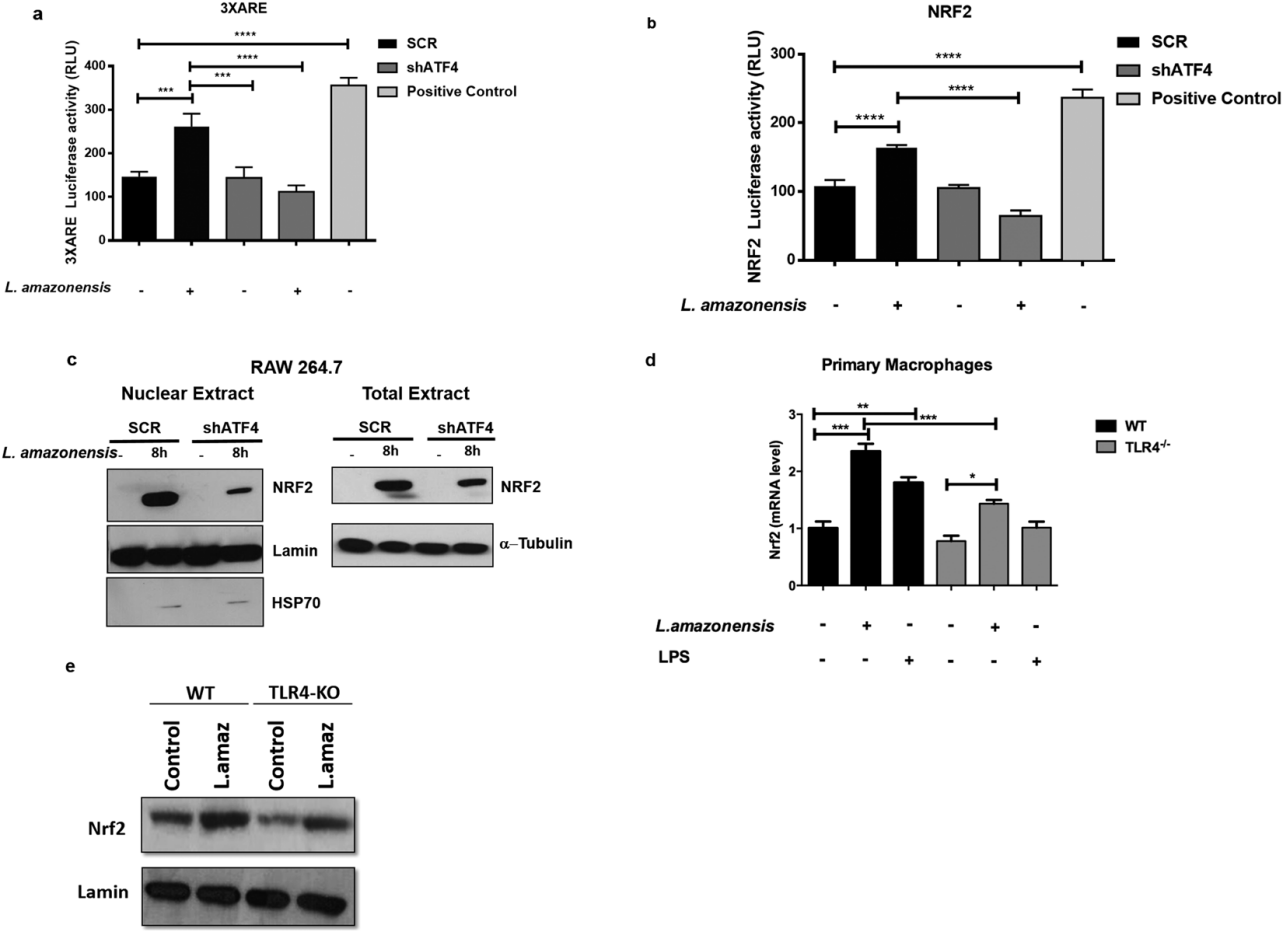
ATF4 controls NRF2 expression and nuclear translocation in *L*. *amazonensis* infected macrophages. (**a**) shATF4 and shSCR cells were transiently transfected with the reporter plasmids containing luciferase ORF under the control of 3XARE consensus site for 24 hours and infected with *L*. *amazonensis* for 24 hours. **indicates p < 0.01; ***indicates p < 0.001. (**b**) shATF4 and shSCR transduced cells were transfected as in B, but with luciferase ORF under the control of NRF2 promoter for 24 hours and infected with *L*. *amazonensis* for 24 hours. *** indicates p < 0.001. (**c**) shATF4 or shSRC transduced cells were infected with *L*. *amazonensis* for 8 hours and total and nuclear extracts were analyzed by western blot with anti-NRF2, a/c-lamin or α−tubulin antibodies. (**d**) Primary macrophages from wild-type or TLR4 knockout mice were infected with *L*. *amazonensis* for 4 hours and the relative expression levels of NRF2 mRNA were estimated by RT-qPCR. *Indicates p < 0.05; **indicates p < 0.01; ***indicates p < 0.001. (**e**) Primary macrophages from wild-type or TLR4 knockout mice were infected with *L*. *amazonensis* for 4 hours and the nuclear extract was analyzed by western blot with anti-NRF2 and a/c-lamin antibodies. Sulforaphane treatment was used as positive control of Nrf2 Luc assyas. Representative western blots are shown from 3 independent experiments.

To determine the role of ATF4 activation on the induction of ARE promoters in the context of the infection, we first carried out luciferase reporter expression under the control of the 3XARE promoter construct. 3XARE elements are anti-oxidant-responsive sequences found at the promoters of ARE-regulated genes. We observed that shATF4- transduced cells infected with *L*. *amazonensis* exhibited decreased 3XARE activity when compared to shSCR-transduced similarly infected cells (Fig. 5a). To understand the role of ATF4 in NRF2 expression, we performed luciferase gene reporter assays with a plasmid construct containing NRF2 promoter sequence displaying ARE elements. We observed that shATF4-transduced cells infected with *L*. *amazonensis* exhibited decreased NRF2-promoter driven luciferase expression compared to shSCR transduced cells infected by *L*. *amazonensis* (Fig. 5b). Sulforaphane (10 μM) was used as a positive control for oxidative stress induction. In addition Western blot analysis of nuclear and total extracts from shSCR or shATF4- infected macrophages show that *L*. *amazonensis* induces the expression and nuclear translocation of NRF2 in ATF4 dependent fashion (Fig. 5c).

As TLR4 is important to ATF4 activation, we tested the role of TLR4 in NRF2- ATF4 dependent expression. Indeed, we observed a reduction in both NRF2 mRNA and protein levels in TLR4 knockout murine primary macrophages compared with the wild type when infected by *L*. *amazonensis* (Fig. 5d,e).

### Human cutaneous leishmaniasis patients display highly ATF4 expression in infected tissue

**Figure 6:**
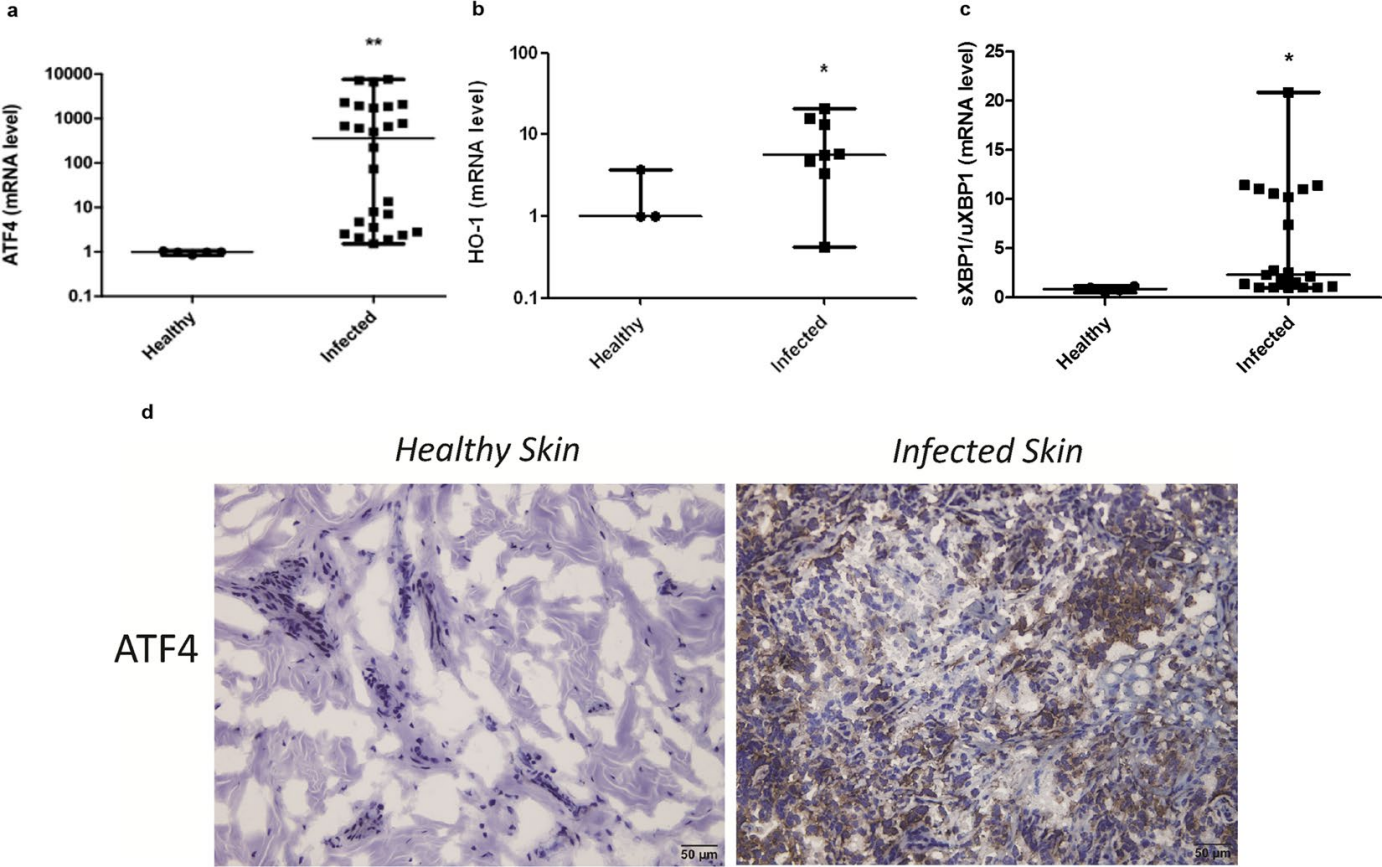
The IERSR and its downstream effectors are induced in patients infected with *L*. *braziliensis*. (**a**) Relative expression levels of ATF4 mRNA were estimated by RT-qPCR using RNA extracted from *L*. *braziliensis* human lesions. ****Indicates p < 0.001. (**b**) Relative expression levels of HO-1 mRNA were measured from *L*. *braziliensis* human lesions as in (**a**). *Indicates p = 0.042. (**c**) The ratio of sXBP1/uXBP1 was measured from RNA samples from *L*. *braziliensis* human lesions by RT-qPCR, as in (**a**). **Indicates p = 0.0089. (**d**) ATF4 protein was visualized in lesions from patients infected with *L*. *braziliensis* by immunostaining samples with anti-ATF4 antibody. Skin biopsies from healthy donors was used as non-infected control. Scale bar: 50 μm.

To determine the relevance of our findings to human leishmaniasis, we compared lesions obtained from patients infected with another prevalent *Leishmania* species that causes CL, with comparable tissue samples obtained from healthy individuals for markers of the IERSR. RT-qPCR assays with RNA samples obtained from skin lesions of *L*. *braziliensis* infected patients or skin biopsies from healthy individuals showed that samples from patients display elevated ATF4 expression (Fig. 6a). Consistently with our previous findings^17^, mRNA levels for XBP1 were also elevated in human infected tissues (Fig. 6c). These data suggest that *Leishmania* parasites activate at least two branches of the IERSR during human infection. Taken together with our demonstration that activation of these two branches of the IERSR is important for *L*. *amazonensis* infection, we concluded that the IERSR may play an important role in CL pathogenicity. If this is correct, then ARE-regulated-genes should be overexpressed in infected human lesions. To test this prediction, we tested HO-1 expression in samples from *L*. *braziliensis* - infected patients that displayed high ATF4 levels. As expected, these samples showed increased HO-1 mRNA expression compared to healthy tissue (Fig. 6b). Finally, in immunohistochemistry assays, biopsy samples from the skin of *L*. *braziliensis* infected patients showed increased ATF4 protein levels when compared with healthy skin obtained from healthy patients (Fig. 6d).

## Discussion

*L*. *amazonensis* belongs to the Mexicana complex and may cause cutaneous and diffuse cutaneous leishmaniasis^2^. These parasites have the unique ability to subvert the host immune response to ensure the progress of the infection, particularly in macrophages. *L*. *amazonensis* disrupts the oxidative stress in macrophages during the infection^3^. This parasite also induces NF-κB homodimer (p50/p50) formation, thereby reducing iNOS expression^18^. Although the pathogenesis in human cutaneous leishmaniasis varies according to distinct *Leishmania* species, viral co-infection and other factors, *L*. *amazonensis* is a widely used research model for studying parasite-host cell interactions^27,28^. Recently, we demonstrated that *L*. *amazonensis* induces IRE1/XBP-1 branch of the IERSR to promote infection both by increasing IFN-β expression and by controlling oxidative stress^16^. In mammalian cells, ER stress triggers the activity of three ER transmembrane proteins: PERK, IRE1, and ATF6. The components of the IERSR are also activated by viral^14^ and bacterial infections, such as *Pseudomonas aeruginosa* and *Brucella melitensis* ^29,30^. Regarding to intracellular parasites, it has been shown that *Plasmodium berghei* infection activates the XBP-1 signaling, which favors infection in the liver^15^. Additionally, it was recently reported that *Leishmania infantum* induces mild ER stress to promote parasite survival^31^. Here, we report that the PERK/eIF2α/ATF4 branch of the IERSR is activated during *Leishmania* infection and that this signaling has an essential role for parasite survival and proliferation. Importantly, we demonstrate that the IERSR and its downstream effector such as the anti-oxidative gene product HO-1 is turned on in human CL lesions.

Phosphorylation of eIF2α by PERK leads to attenuation of overall translation, while increasing translation of a subset of mRNAs with multiple tandem uORFs in their 5′UTR, including ATF4 mRNA^21,22^. ATF4 induces transcription of genes governing ER homeostasis, autophagy and oxidative stress response^10,11^. Our data revealed that PERK/eIF2α/ATF4 signaling is required for *Leishmania*-induced expression of the anti-oxidative response gene HO-1. Importantly, uninfected shATF4 transduced macrophages exhibit increased NO production, therefore emphasizing the role of ATF4 in the modulation of the oxidative milieu.

We have previously demonstrated that *L*. *amazonensis* activates the dsRNA-activated kinase (PKR), which is another eIF2α kinase^4^. Our present data support the importance of eIF2α phosphorylation for the success of the infection by at least these two kinases, leading to the expression of downstream effectors that act, probably in conjunction, to damper some macrophage functions.

Our data demonstrate that ATF4 plays an important role in the activation of ARE promoters by *L*. *amazonensis*. Consistently, shATF4 transduced cells infected with *L*. *amazonensis* display reduced transcriptional activation of NRF2. NRF2 is an important anti-oxidant transcriptional factor that binds to ARE elements to induce the expression of anti-oxidant genes such as HO-1. HO-1 reduces oxidative stress, removing toxic heme and billiverdin products, iron ions and carbon monoxide^32^. HO-1 plays an important role in the infection by *Leishmania* parasites, as previously described for *Leishmania* chagasi^33^. We have previously demonstrated that the IRE1/XBP-1 branch of the IERSR plays an important role in *L*. *amazonensis* induced HO-1 expression^16^. Here we show that ATF4 induced by *L*. *amazonensis* infection correlates with increased HO-1 expression (Fig. 4). It is conceivable that ATF4 and NRF2, both induced by *L*. *amazonensis* infection, collaboratively induces the HO-1 expression given the role of ATF4 in NRF2 (Fig. 5). ATF4 nuclear translocation is dependent on TLR4 that plays a role in *L*. *amazonensis* infection (Fig. 3). Importantly, our data show that *L*. *amazonensis* growth was reduced in infected TLR4KO macrophages and the addition Thapsigargin did not enhance the intracellular load of this parasite. Our together, these results indicate the role of TLR4 in the PERK/eIF2α/ATF4 signaling triggered by *L*. *amazonensis*. Acordingly, NRF2 activation is partially dependent on TLR4 (Fig. 5). Recent work of our group showed that NRF2 is critical for the anti-oxidative host response triggered during *Leishmania* infection, being dependent on PKR activation^34^.We further demonstrated that activation of anti-oxidative cell response promotes *L*. *amazonensis* infection in macrophages, as addition of an anti-oxidant compound (NAC) to shATF4 transduced cells full restores the infection potential.

To address the clinical relevance of our *in vitro* mechanistic data, we assayed expression of ATF4, XBP-1 and HO-1 in clinical samples obtained from patients with cutaneous leishmaniasis. Remarkably, most lesions showed elevated RNA levels for ATF4, XBP-1 and HO-1. Immunohistochemistry analysis confirmed increased ATF4 protein expression in clinical samples. These results support our hypothesis that IERSR is activated in human tissue infected by *Leishmania* and may play a critical role in the pathogenesis. Accordingly, ER stress may shape some inflammatory processes due to the production pro-inflammatory cytokines, NF-kB activation and formation of NLRP3 inflamasome^35–38^. It is conceivable that TLR4 ligands expressed by the *Leishmania* parasites may act in conjunction with other factors, such as the formation of parasitophorous vacuole developed during the infection and the constant recruitment of ER components to it, to trigger IERSR^39,40^. All the clinical samples analyzed in this study were derived from *L*. *braziliensis* infections, the most prominent causative agent of cutaneous leishmaniasis in Brazil. Although *L*. *amazonensis* and *L*. *braziliensis* present remarkable differences in pathogenesis, our results indicate that the activation of the IERSR downstream effector ATF4, and the expression of ARE promoter driven genes such as HO-1 may represent conserved evasive mechanism shared by at least these two *Leishmania* species. (Fig. 6).

**Figure 7:**
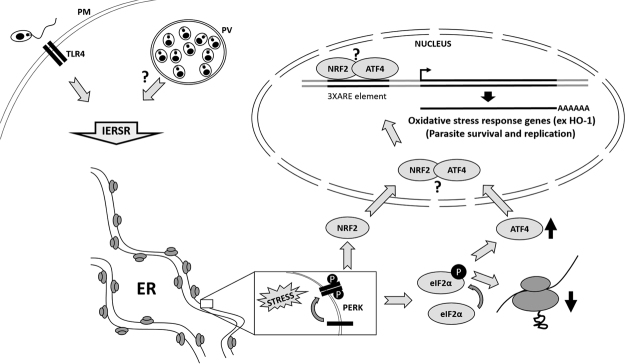
Schematic model for the role of PERK/eIF2α/ATF4 in leishmaniasis. The PERK/eIF2α phosphorylation/ATF4 axis suppresses translation initiation to reduce the demand on protein folding while inducing the expression of genes required for restoring ER-homeostasis. ATF4 also induces expression of genes that play critical role in resistance to oxidative stress. Our data demonstrates that *Leishmania* parasites activate the PERK/eIF2α/ATF-4 pathway and that this pathway is important for parasite survival and pathogenesis. PM: plasma membrane; PV parasitophorous vacuole; interrogation marks [?] point to unsolved questions, discussed in the text.

 Figure 7 presents a schematic model summarizing our observations and indicating some questions to be addressed in the future. Therefore, the IERSR emerges as a potential target for chemotherapeutic development and clinical intervention in cutaneous leishmaniasis.

## Electronic supplementary material

Supplementary Information
