# Supplementary material for: Emerging Role for the PERK/eIF2α/ATF4 in Human Cutaneous Leishmaniasis
Source: Sci Rep. 2017 Dec 6;7:17074. doi: 10.1038/s41598-017-17252-x (PMC5719050; doi:10.1038/s41598-017-17252-x)
Supplement: Supplementary file 1 — Supplementary Information [file 41598_2017_17252_MOESM1_ESM.pdf]

Emerging Role for the PERK/eIF2α/ATF4 in Human Cutaneous Leishmaniasis

Karina Luiza Dias-Teixeira[a,b], Teresa C. Calegari-Silva[a], Jorge M. Medina[c], Áislan C. Vivarini[a], Átila Cavalcanti[a],Nataly Teteo[a], Alynne Karen M. Santana[d], Fernando Real[e], Ciro M. Gomes[f], Renata Meirelles Santos Pereira[g], Nicolas Fasel[h], João S. Silva[d], Bertal H. Aktas[b], Ulisses G. Lopes[a,*].

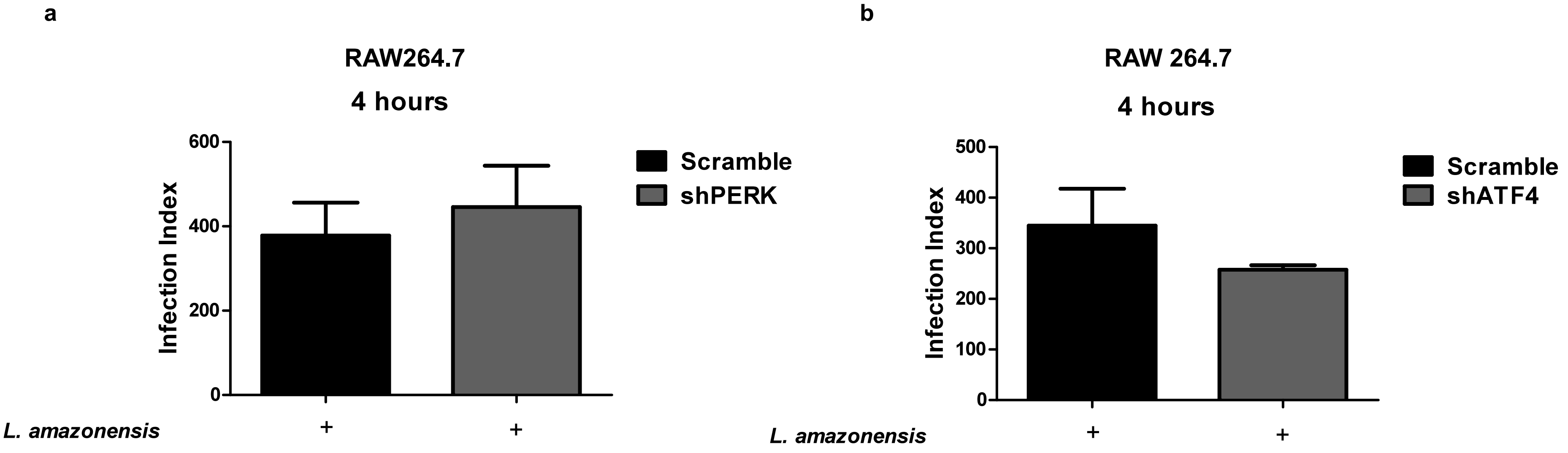

Supplementary figure 01. Depletion of PERK and ATF4 reduces *L. amazonensis* infection by interfering not with entry, but with parasite survival inside macrophages. A) shSCR and shPERK transduced RAW 264.7 cells were infected with *L. amazonensis* for 4, fixed and stained with Giemsa. B) shSCR and shATF4 transduced RAW 264.7 cells were infected with *L. amazonensis* for 4, fixed and stained with Giemsa as in (A). Infection Indexes was measured as percent of infected cells × number of the amastigotes/cell.
